# COVID-19 Encephalopathy in Adults

**DOI:** 10.7759/cureus.13052

**Published:** 2021-02-01

**Authors:** Paavani Atluri, Deepa Vasireddy, Srikrishna Malayala

**Affiliations:** 1 Internal Medicine, Bay Area Hospital, Coos bay, USA; 2 Pediatrics, Pediatric Group of Acadiana, Lafayette, USA; 3 Internal Medicine, Temple University Hospital, Philadelphia, USA

**Keywords:** covid-19, encephalopathy, sars-cov-2 virus, cognitive impairment in covid-19

## Abstract

Many patients with COVID-19 are asymptomatic. However, among the patients that are symptomatic, influenza-like illnesses including fever, myalgia and respiratory symptoms seem to be the most common presentation across age groups. Though respiratory illness seems to be the primary presentation, about 36.4% to 69% of hospitalized COVID-19 patients have exhibited neurological manifestations.

We present two patients who were hospitalized for the presenting symptom of acute encephalopathy. Both the patients regained consciousness within 24 to 48 hours of initiating treatment. The first patient was known to have mild cognitive impairment and a thorough work-up was done in the emergency department which did not reveal any other causes apart from positive SARS-CoV-2 rapid PCR test. The second patient was from a long-term care facility with underlying dementia, usually alert, awake and oriented to self and presented with severe encephalopathy with a Glasgow Coma Scale of 3 on admission. Her work up was notable only for a positive SARS-CoV-2 rapid polymerase chain reaction test. Both patients responded well to standard remdesivir and steroid therapy and returned to baseline cognition.

SARS-COV 2 virus appears to be a causative agent of acute onset encephalopathy. Very little is known about the pathophysiology of neurological manifestations in COVID-19 illness. There are several theoretical possibilities of pathogenesis such as of blood-brain barrier disruption secondary to SARS-CoV-2 binding to angiotensin-converting enzyme 2, autoimmune sequelae, ischemic injury via systemic hypoxia or local vascular endothelial information or thrombosis, toxic metabolic encephalopathies and long-term impact of systemic proinflammatory state that have been considered.

## Introduction

SARS-CoV-2 virus is the main pathogen for the COVID-19 pandemic and the scale and speed of the pandemic is unprecedented with limited available data, knowledge, and resources to contain the pandemic. In one meta- analysis of 47 studies on seroprevalence covering 399,265 people from 23 countries, the SARS-CoV-2 prevalence in the general population varied from 0.37% to 22.1%, with the pooled estimate of 3.38% [1]. The origin of the virus is suspected to be from animal to human. The initiating event is still being investigated and the transmission is primarily human to human. The virus was initially recognized in December 2019 by Chinese authorities in the setting of cases of pneumonia that seem to be clustered around a seafood market in Wuhan, Hubei province [2-4].

Majority of the patients are asymptomatic and in those that are symptomatic, most present with influenza-like illnesses but many do not present with the classic combination. Some present with less usual findings such as perniosis or anosmia. These ranges are pulled from many studies and symptom prevalence varies greatly depending on testing and survey methodology.The common presentations are fever according to 44-94%, cough noted in 68-83%, anosmia and/or ageusia in close to 70%, upper respiratory symptoms including sore throat, rhinorrhea, nasal or sinus congestion in 5-61%, shortness of breath in 11-40%, fatigue in 23-38%, muscle aches 11-63%, headache in 8-14%, confusion in 9%, GI symptoms including nausea vomiting and diarrhea in 3-17% [2, 5-15] . About 97.5% of exposed cases will develop symptoms within 11 days and 99% within 14 days. Incubation periods of up to 24 days are shown in some reports but mean and median incubation time is 5 days with the common range of 2-7 days [2, 5, 7-8, 16].

## Case presentation

Case 1

A 86-year-old female with known history of hypertension, hyperlipidemia, history of colon cancer, significant physical deconditioning-wheelchair dependent, history of pressure ulcers in the past, calcified atherosclerosis of abdominal aorta. incidental finding of meningioma noted on the Computerized tomography (CT) scan head on admission who is known to have moderate cognitive impairment at baseline was sent from long-term care facility to the emergency department for unresponsiveness, fever of 101 °F, tachypnea and tachycardia. Patient was recently diagnosed with acute E. coli urinary tract infection (UTI) 4 days prior to the admission and was treated with trimethoprim / sulfamethoxazole for 5 days per identification and susceptibilities result.

Patient was sent to the emergency department given worsening encephalopathy along with fever, tachypnea and tachycardia for further evaluation. The admitting physician could not elicit a history from the patient as she appeared to be very confused and was lethargic but easily arousable to speech. According to the staff at the long-term care facility, the patient did not have a cough, chronic hypoxic respiratory failure, abdominal pain, dizziness, nausea, episodes of emesis. Her last bowel movement was 3 days prior to the admission. She had urinary incontinence at baseline. Patient was not able to comment on anosmia or dysgeusia/ageusia on admission. Patient was able to maintain saturations around 95-96% with an oxygen support of 0.5 to 1 L/min via nasal cannula. She was also able to maintain airway and did not require positive pressure ventilation or mechanical ventilation at any point during her hospitalization stay.

A thorough work-up was done in the emergency department and laboratory work up of note revealed leukocytosis with neutrophilia, hypernatremia, hyperchloremia, metabolic acidosis without elevated anion gap, hyperglycemia , elevated lactate dehydrogenase (LDH), transaminitis, elevated troponin which was thought to be type II myocardial infarction secondary to rapid ventricular rate with a flutter being the underlying rhythm, elevated C reactive protein (CRP), elevated procalcitonin, elevated D-dimer and elevated NT-proBNP (Table 1). 

**Table 1:** Laboratory findings of Case 1 on day of admission

Laboratory parameters	Reference range	Day of admission (Patient 1)
White blood cell count	(4-11) X 10^3^/𝜇L	13.1
Hemoglobin	(12-15.5) g/DL	14.3
Platelets	(140-425)X 10^3^/𝜇L	241
Absolute Neutrophil count	(1.5-7.5) X 10^3^	10.8
Absolute Lymphocyte count	(1-4)X 10^3^	1.2
Absolute Monocyte count	(0.2-1.0) X 10^3^	0.9
Erythrocyte Sedimentation rate	(0-30) mm/hr	18
C Reactive Protein	(<1 mg/dl)	3.458
NT-proBNP	(11.1- 449.9)pg/ml	15,300
D-dimer	(<=500)ng/ml	6828
Protime	(9.4-12.5) seconds	15.7
INR	(0.9-1.1)	1.3
Troponin -1	(<0.04)ng/ml	0.12
Ferritin	(11-307)ng/ml	151
Procalcitonin	(<0.05)ng/ml	0.07
Sodium	(135-145) mmol/L	147
Potassium	(3.5-5.0)mmol/L	4.2
Chloride	(100-110)mmol/L	120
CO2 level	(21-31)mmol/L	17
Anion gap	(7-17) mmol/L	14
Glucose	(70-106) mg/dl	119
BUN	(8-26) mg/dl	31
Creatinine	(0.5-1.2) mg/dl	0.88
AST	(10-50) U/L	60
ALT	(<34)U/L	42
Alkaline Phosphatase	(42-121) U/L	72
LDH	(313-618) U/L	804
Creatinine Kinase	(22-269) U/L	25

Lactic acid was checked on admission and noted to be normal at 1.5 mmol/L. An arterial blood gas was obtained which did not show any hypoxic or hypercarbic respiratory failure. Urinalysis was repeated on admission which was not indicative of an infection. Two sets of blood cultures were drawn in the emergency department which did not grow any organisms. A SARS-CoV-2 rapid polymerase chain reaction (PCR) test was done in the emergency department which was noted to be positive. 

**Figure 1:**
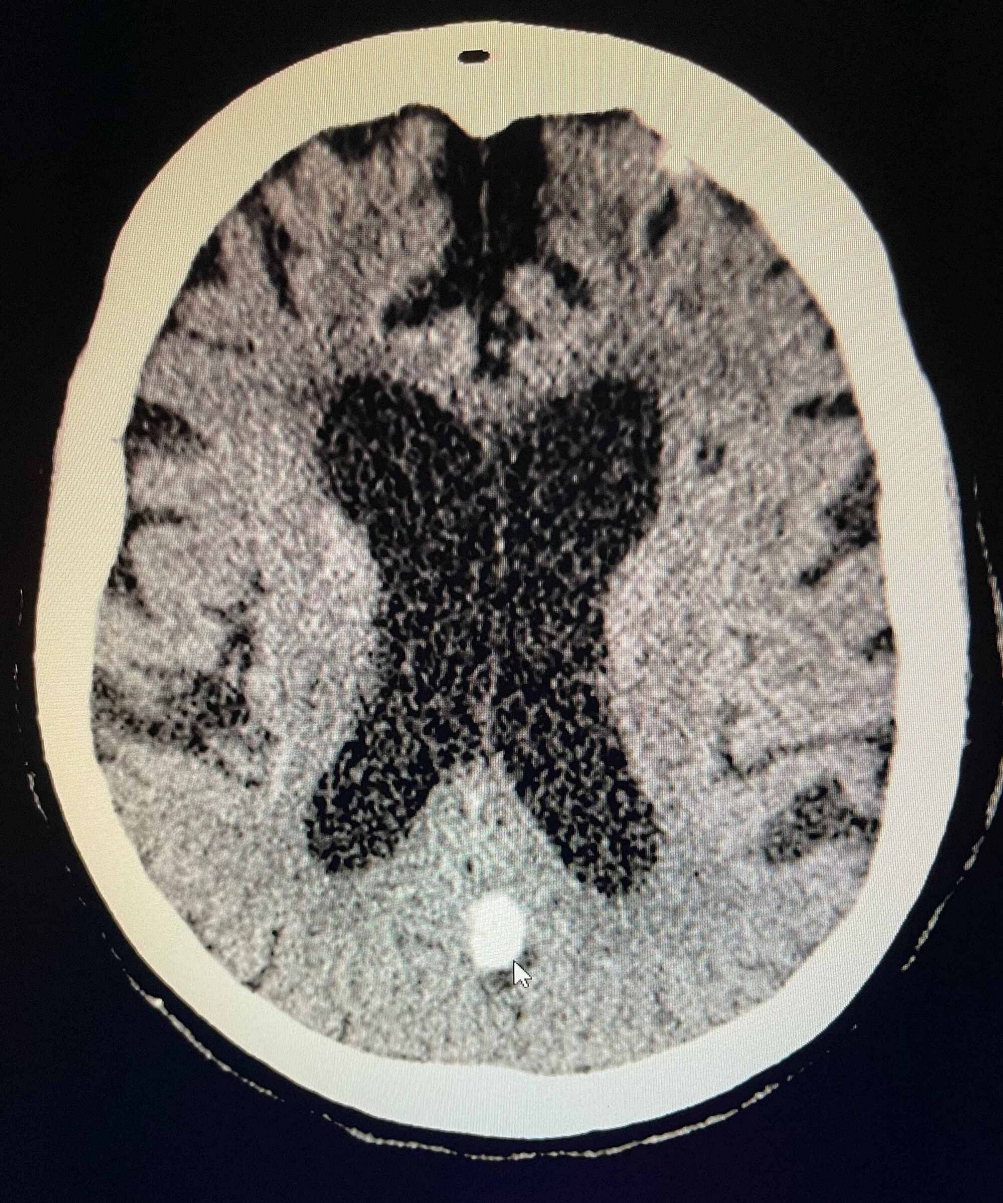
Computerized tomography (CT) scan of the head showing volume loss with disproportionate involvement of medial temporal lobes and a 12 mm calcified probable meningioma of left parafalcine region.

To further assess for encephalopathy, a computerized tomography scan of the head without contrast was done which showed no evidence of acute abnormality, volume loss with disproportionate involvement of medial temporal lobes which can be associated with Alzheimer’s dementia and frontotemporal lobar degeneration. A 12 mm calcified probable meningioma of left parafalcine region was also incidentally noted (Figure 1).

CT of the abdomen and pelvis with IV contrast showed moderate left and small right pleural effusions and partial atelectasis of left lower lobe with no evidence of acute abnormality within abdomen. However, there was motion artifact degrading evaluation. A chest x-ray was done on admission which showed retrocardiac opacity probably representing partial left lower lobe atelectasis; pneumonia could also have this appearance per radiology read. Normal cardiomediastinal contours were noted. Patient was admitted to an intermediate medical care unit with telemetry and she was empirically started on Piperacillin-Tazobactam 3.375 g every six hours which was deescalated to Amoxicillin / Clavulanic acid on day 4. She was also started on Dexamethasone 6 mg IV daily and remdesivir course-given as 200 mg on day 1 followed by 100 mg daily for 4 days. An echocardiogram performed to further assess systolic and diastolic function given elevated proBNP on admission showed moderate LV dysfunction with moderate and diffuse left ventricle (LV) hypokinesis with ejection fraction of 35 to 40%, moderately depressed right ventricle (RV) free wall motion, severe left atrial and right atrial enlargement, mildly thickened aortic valve without aortic stenosis and aortic regurgitation, moderate mitral regurgitation with mildly elevated calculated right ventricular systolic pressures with no prior echocardiograms available for comparison. Within 48 hours, the patient was fully awake, responding to commands, was able to tell her name and date of birth and could not recognize her daughter’s name, but was not oriented to time, place or situation. Power was 5/5 in all the extremities and no gross motor or sensory deficits were appreciated. According to the family, the patient did have moderate to severe cognitive impairment and has significant short-term memory loss. We were not able to identify any other infectious or metabolic source to explain the patient's encephalopathy. She had remained hemodynamically stable throughout the course until day 6 of admission when a rapid response was called for an episode of hypotension and worsening acute respiratory failure. At that time, the family had decided to keep the patient comfortable and the patient eventually expired on day 12 of admission.

Case 2

A 85-year-old female with known history of anxiety disorder, coronary artery disease, diabetes, hypertension, hyperlipidemia, coronary artery disease, cerebrovascular accident (CVA) and chronic anemia was sent to the emergency department by the family for acute encephalopathy. Patient was diagnosed with a UTI 3 weeks prior to presenting to the emergency department and finished a course of antibiotics but was still feeling weak. Hence, a chest x-ray was done by the primary care provider during the follow- up appointment which showed findings concerning for pneumonia and a CT of the chest was done along with a SARS-CoV-2 PCR test. Patient had a positive SARS-CoV-2 PCR test 2 days prior to presenting to the emergency department. Patient had mild cognitive impairment at baseline as noted by the family and was currently being worked up by the primary care provider as an outpatient. According to the patient’s daughter, they were able to notice some personality changes going on for at least 4 to 5 months prior to her admission. Patient was alert and awake but was not oriented to time, place, person and situation. She was not in any acute cardiorespiratory distress. She denied to have fever, chills, chest pain, chest pressure, shortness of breath, abdominal pain, nausea, recent changes in bowel or bladder habits. She was only able to recall her first name and could not recall her last name or date of birth. A thorough investigation was done in the emergency department and laboratory work up of note showed hyponatremia, hyperglycemia, elevated CRP,elevated erythrocyte sedimentation rate (ESR),elevated procalcitonin, microcytosis with hypochromia with mild anemia and thrombocytosis and elevated D-dimer (Table 2).

**Table 2:** Laboratory findings of Case 2 on day of admission

Laboratory parameters	Reference range	Day of admission (Patient 2)
White blood cell count	(4-11) X 10^3^/𝜇L	7.4
Hemoglobin	(12-15.5) g/DL	11.8
Platelets	(140-425)X 10^3^/𝜇L	486
Absolute Neutrophil count	(1.5-7.5) X 10^3^	5.6
Absolute Lymphocyte count	(1-4)X 10^3^	0.8
Absolute Monocyte count	(0.2-1.0) X 10^3^	0.8
Erythrocyte Sedimentation rate	(0-30) mm/hr	51
C Reactive Protein	(<1 mg/dl)	5.531
D-dimer	(<=500)ng/ml	1895
Ferritin	(11-307)ng/ml	377
Procalcitonin	(<0.05)ng/ml	0.12
Sodium	(135-145) mmol/L	134
Potassium	(3.5-5.0)mmol/L	4
Chloride	(100-110)mmol/L	100
CO2 level	(21-31)mmol/L	25
Anion gap	(7-17) mmol/L	13
Glucose	(70-106) mg/dl	238
BUN	(8-26) mg/dl	14
Creatinine	(0.5-1.2) mg/dl	1.06
AST	(10-50) U/L	22
ALT	(<34)U/L	11
Alkaline Phosphatase	(42-121) U/L	77
LDH	(313-618) U/L	444
Creatinine Kinase	(22-269) U/L	26

**Figure 2:**
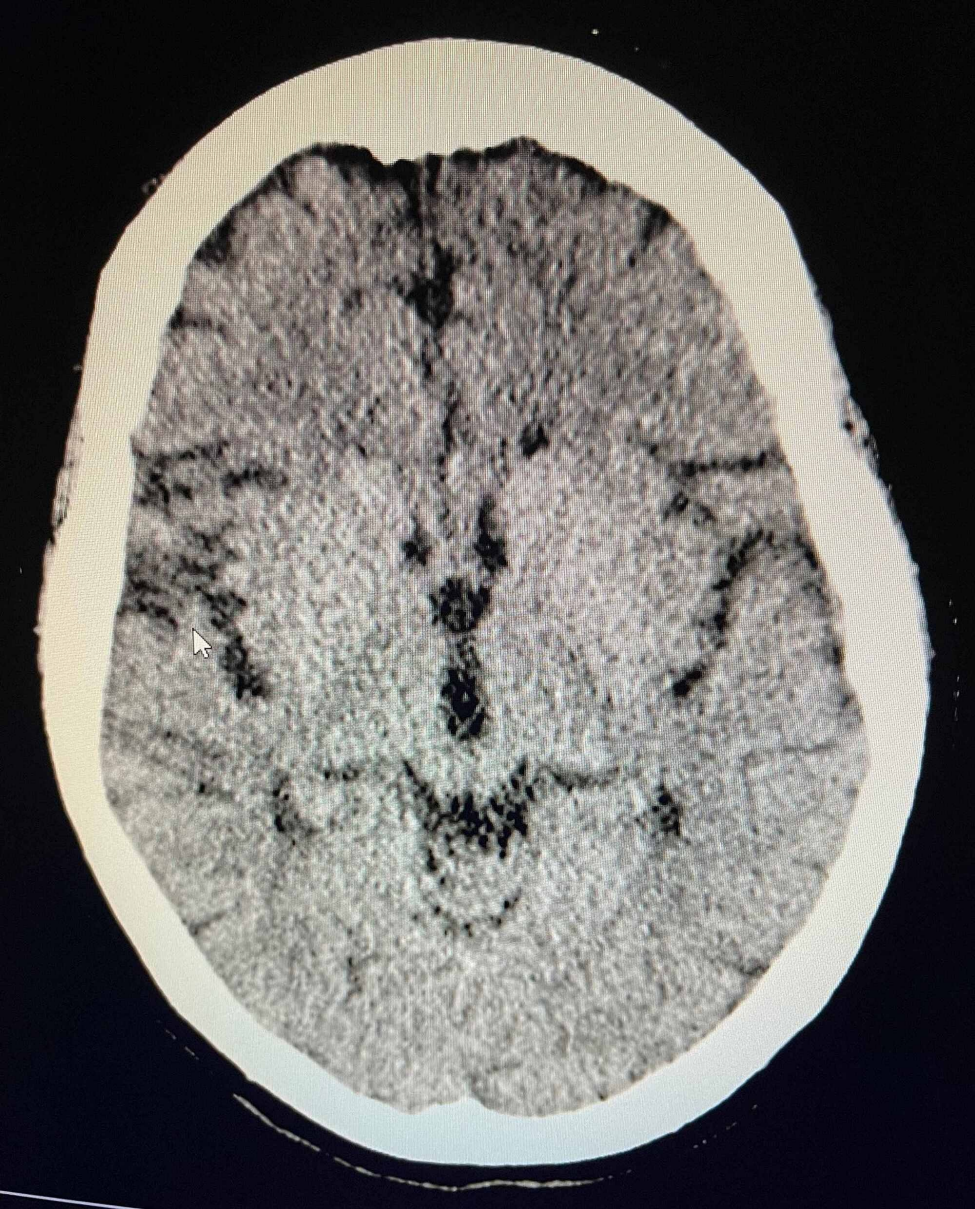
Computerized tomography (CT) scan of the head showing cortical atrophy and mild chronic small vessel white matter ischemic disease and an old infarct in the right posterior parietal lobe.

Urinalysis was done in the emergency department which did not indicate an infection. CT of the head without contrast showed no acute intracranial process, cortical atrophy and mild chronic small vessel white matter ischemic disease; old infarct in the right posterior parietal lobe was noted which was present on her previous CT scan as well (Figure 2).

Chest x-ray was done in the emergency department which showed interstitial opacities throughout the lungs (left greater than right) suggesting an atypical infectious/inflammatory process with mild cardiomegaly. Patient was admitted to the hospital and was started on remdesivir course: 200 mg on day 1 followed by 100 mg daily for 4 days and Decadron 6 mg daily. Given the patient’s confusion, consent to start remdesivir therapy was obtained from the patient's daughter. She was also started on supportive treatment and the rest of her home medications were continued. Patient’s acute encephalopathy was resolved within 24 hours and on repeated neurological evaluations, was noted to be alert, awake, oriented x4 with no gross motor or sensory neurological deficits. Physical therapy was consulted and she finished a course of remdesivir and was discharged home in a stable condition with down trending inflammatory markers.

## Discussion

We present here 2 cases of COVID-19 whose major presentation was acute severe encephalopathy which had resolved within 24 to 48 hours of treatment initiation. An extensive work-up was done on admission to rule out toxic, metabolic causes and other interacting effects of multiple comorbidities to delineate the etiology of acute severe encephalopathy. However, none were identified and hence we conclude that the encephalopathy is the primary presentation of COVID-19 illness. Based on the acuity of presenting symptoms, history and clinical signs, symptoms and laboratory work-up, there are several different causative factors of encephalopathy for the clinician to consider (Table 3).

**Table 3:** Etiological factors for encephalopathy

Toxic	Medications, illicit drugs and toxic chemicals (lead, mercury and arsenic)
Metabolic	Hepatic or renal failure, dehydration, electrolyte imbalance, Thiamine deficiency (Wernicke encephalopathy)
Trauma	Physical injury (acute or as chronic traumatic with repetitive injury)
Infectious	Most bacterial and viral illnesses. Rare causes- Prion diseases, Lyme disease associated encephalopathy, Salmonella associated encephalopathy
Hereditary	Mitochondrial encephalopathy
Autoimmune	Hashimoto’s encephalopathy
Systemic	Hypertension, hypotension, hypoxia, epilepsy induced encephalopathy.

We did struggle to understand the pathophysiology of encephalopathy in these patients as very little is known about neurological manifestations in COVID-19 illness. Though respiratory illness seems to be the primary presentation in 36.4% to 69% of hospitalized COVID-19 patients [17-18], neurological manifestations were noted. The known neurological manifestations of COVID-19 are delirium, confusion or executive dysfunction, smell or taste abnormalities, headache, corticospinal tract signs, dizziness, stroke, Guillain-Barre syndrome, vestibular neuritis, hemifacial spasms, Miller Fisher syndrome and demyelinating lesions [17-20]. 

The pathophysiology of various neurological manifestations of COVID-19 remains largely unknown. The possible mechanisms from literature review include direct viral invasion of the nervous system with potential trans-synaptic spread, blood-brain barrier disruption secondary to SARS-CoV-2 binding to angiotensin-converting enzyme 2, autoimmune sequelae, ischemic injury via systemic hypoxia or local vascular endothelial information or thrombosis, toxic metabolic encephalopathies and the long-term impact of systemic proinflammatory state. Based on the analysis by Liotta et al., encephalopathy seems to be the third most frequently observed neurological manifestation-correlated independently with worsening functional outcomes and greater mortality. 

## Conclusions

The long-term sequelae of neurological manifestations in COVID-19 cases remains unclear at this time. Both our patients did not have any signs of meningoencephalitis warranting invasive investigations like lumbar puncture, but it should be definitely considered in patients who meet the criteria. We hope our cases generate further discussions about neurological manifestations in COVID-19 cases helping us better understand the pathophysiology and short-term and long-term sequelae.
